# The Influence of *Staphylococcus aureus* on Gut Microbial Ecology in an *In Vitro* Continuous Culture Human Colonic Model System

**DOI:** 10.1371/journal.pone.0023227

**Published:** 2011-08-09

**Authors:** Thippeswamy H. Sannasiddappa, Adele Costabile, Glenn R. Gibson, Simon R. Clarke

**Affiliations:** 1 School of Biological Sciences, University of Reading, Reading, United Kingdom; 2 Food Microbial Sciences Unit, School of Food and Nutritional Sciences, University of Reading, Reading, United Kingdom; Columbia University, United States of America

## Abstract

An anaerobic three-stage continuous culture model of the human colon (gut model), which represent different anatomical areas of the large intestine, was used to study the effect of *S. aureus* infection of the gut on the resident faecal microbiota. Studies on the development of the microbiota in the three vessels were performed and bacteria identified by culture independent fluorescence *in situ* hybridization (FISH). Furtheremore, short chain fatty acids (SCFA), as principal end products of gut bacterial metabolism, were measured along with a quantitative assessment of the predominant microbiota. During steady state conditions, numbers of *S. aureus* cells stabilised until they were washed out, but populations of indigenous bacteria were transiently altered; thus *S. aureus* was able to compromise colonisation resistance by the colonic microbiota. Furthermore, the concentration of butyric acid in the vessel representing the proximal colon was significantly decreased by infection. Thus infection by *S. aureus* appears to be able to alter the overall structure of the human colonic microbiota and the microbial metabolic profiles. This work provides an initial *in vitro* model to analyse interactions with pathogens.

## Introduction

*Staphylococcus aureus* is a major opportunistic human pathogen, able to cause a wide variety of disease in humans and animals. It is the cause of a large burden of morbidity and mortality, globally, in both hospital and community settings [1]. Despite the plethora of antimicrobial agents available, infection continues to spread and many strains are resistant to an array of antibiotics. In particular, methicillin resistant *S. aureus* (MRSA) is an acute clinical problem, with many hospital and community isolates displaying resistance [1]. In response, measures have been developed to prevent the spread of this naturally ubiquitous organism, which lives as a commensal in the nares of 20–25% of the population at any one time [2], [3]. While nasal colonisation is a well-established risk factor for most types of infections, several recent studies have suggested that colonisation of the intestines, which occurs in *c*. 20% of individuals and which relatively, has been less intensively studied, could have important clinical implications [4]. Patients with *S. aureus* intestinal colonisation may serve as an important source of transmission, as they often contaminate the adjacent environment [5]. Factors such as faecal incontinence and diarrhea contribute to dissemination of the pathogen in the healthcare environment [6]. Similarly, such patients display increased frequencies of skin colonisation [7] and studies in intensive care and liver transplant units have shown that patients colonised by MRSA at the rectum and nares have a significantly higher risk of disease than patients with nasal colonisation alone [8]. Furthermore, hospitalised patients reported co-colonisation by *S. aureus* and vancomycin-resistant enterococci in >50% of the individuals studied [6]. Thus, intestinal colonisation by *S. aureus* provides the pathogen with the opportunity to acquire new antibiotic resistance genes.

The clinical implications of intestinal colonisation by *S. aureus* are still relatively ill-defined. It is assumed that carriage imposes a risk for intestinal infection. *S. aureus* can induce psuedomembranous colitis that is histologically distinct from that caused by *Clostridium difficile* [9]. Several studies have shown that intestinal colonisation occurs frequently in infants, particularly those that have been breast-fed [10] and is positively correlated with development of allergies and increased expression levels of the soluble immune modulator CD14, in such children [11]–[15]. The role of intestinal carriage in development of systemic *S. aureus* disease has not yet been established. However in mice, colonisation of the intestinal lumen can lead to *S. aureus* crossing the intestinal epithelial barrier and subsequent spread to the mesenteric lymph nodes [16], [17].

The human intestine represents a complex ecosystem with many commensal microflora species, various types of secretory fluids, fermentation metabolites of digested food and host defense molecules [18]. Survival and subsequent successful colonisation of pathogenic bacteria in the human intestinal tract requires them to resist innate defenses in the intestine. Colonisation resistance by normal commensal bacteria, acidic pH, fermentation metabolites like short chain fatty acids (SCFA), high osmolarity, local gut mucosal immunity by host defense molecules and bile acids in the intestine represent major obstacles for the survival and colonisation of invading pathogenic bacteria.

To date, no suitable *in vivo* models have been used to study carriage and survival of *S. aureus* in the human intestine. Laboratory mouse models of infection do not reproduce the complex microbial ecosystem or the physicochemical environment of the human gut [19].

An *in vitro* three-stage continuous culture system (gut model) is a useful tool to monitor the ecology and metabolic activities of the microbiota in the proximal, transverse and distal colon, in particular in relation to different environmental conditions, dietary intervention, as well as administration of drugs and antimicrobials [20], [21]. In this study, we investigated the impact of *S. aureus* on the normal intestinal flora, and survival of the pathogen, using a three-stage continuous culture model system of the human colon. We found that infection had a significant impact on the normal colonic microflora. Futhermore, short chain fatty acids (SCFA) generation, end products of gut bacterial metabolism, were measured and found to be altered significantly by the presence of *S. aureus*. Thus *S. aureus* is able to influence the composition in the human gut, of both the microbiota and SCFAs.

## Results

### Survival of *S. aureus* in a continuous culture model of the human gut

**Figure 1:**
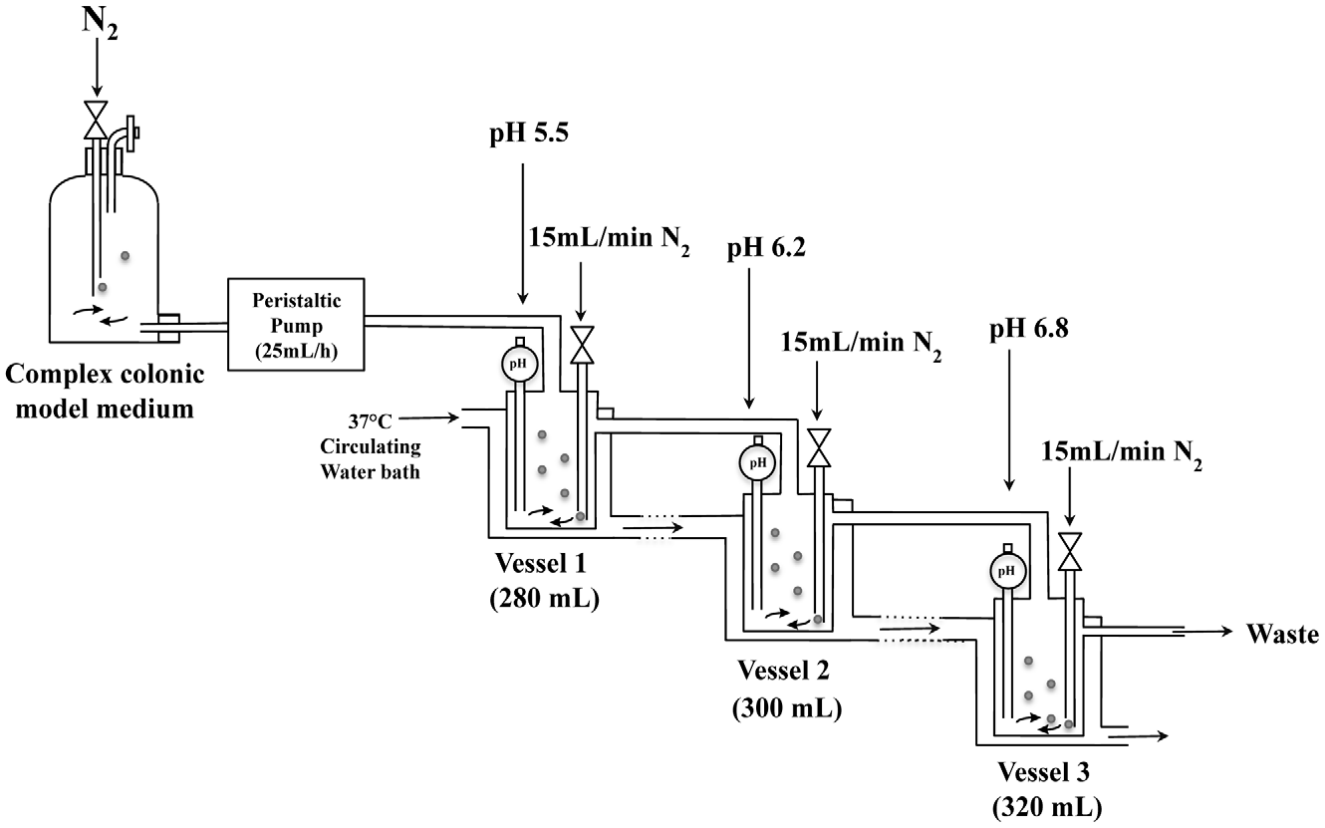
Schematic diagram of the *in vitro* three-stage culture colonic model system (human colonic model). Each vessel was continually sparged with O_2_ free N_2_. The pH of each vessel was individually maintained via automated addition of 1M HCl or 1M NaOH, as required. The system was maintained at 37°C and stirred continuously.

**Figure 2:**
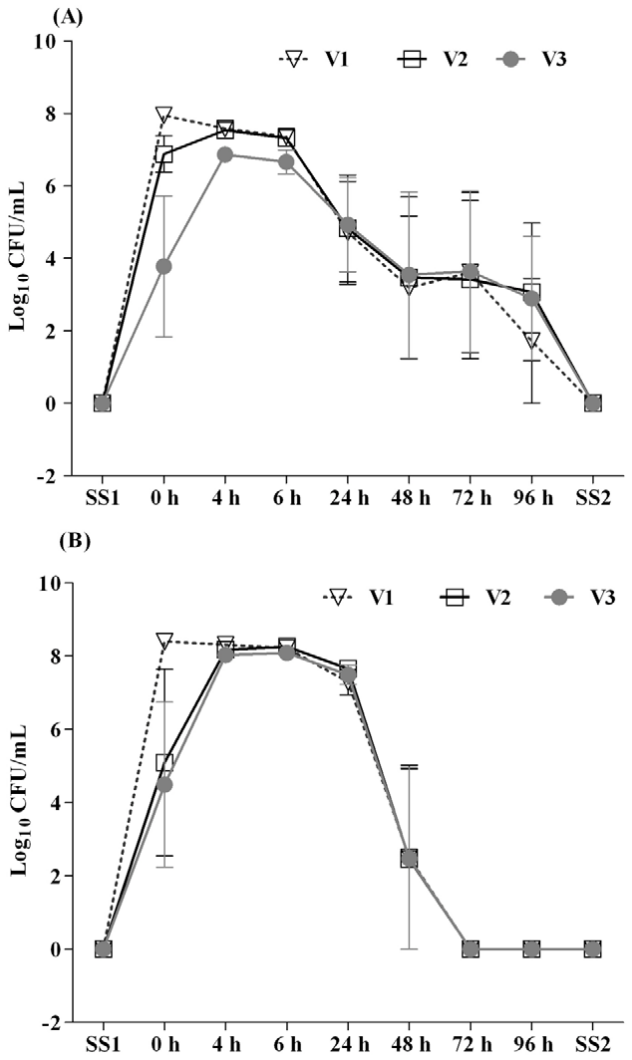
*S. aureus* populations detected in culture broths recovered from the three different vessels (V1, V2 and V3) of the colonic model before (SS1) and after (0, 4, 6, 24, 48, 72, and 96 h and SS2) inoculation of *S. aureus* by viable plate count (A) and FISH method (B). Results are reported as means (Log_10_ CFU/mL) of the data of three colonic models ± standard error of mean. The apparent

An *in vitro* three-stage culture system was used to model the conditions found in the human colon (Figure 1). Post-infection survival of *S. aureus* in three independent *in vitro* colonic models was quantified by viable colony count methods using selective BHI; all colonies displayed identical morphology. Prior to *S. aureus* inoculation into V1 of each colonic model, colony counts at SS1 revealed absence of detectable *S. aurues* in the colonic models (Figure 2A). After inoculating the colonic models with *S. aureus* to a concentration of *c.* 2×10^10^ CFU/mL, as a single dose, the *S. aureus* populations stabilised at 6 to 7 Log_10_ units over a period of 4 and 6 h in all the three vessels of colonic models (Figure 2A). Sub populations of 3 to 4 Log_10_ units were found over a period of 24, 48, and 72 h in all three vessels and less than 2 to3 Log_10_ units counts were found at 96 h after initial inoculation (Figure 2A). No *S. aureus* was detected in any of the three vessels at SS2.

*S. aureus* populations were also quantified by FISH, using a Cy3 labelled-16S rRNA targeted oligonucleotide probe specific to *S. aureus*. Enumeration of *S. aureus* populations by FISH revealed similar patterns of survival in all the three vessels compared to the viable colony count method, although some differences occur due to the higher threshold of detection of FISH. The absence of *S. aureus* from the faecal samples used, was confirmed prior to the inoculation of *S. aureus* into the colonic models at SS1. After the initial inoculation, *S. aureus* achieved stabilsing populations of 7 to 8 Log_10_ units over a period of 4, 6, and 24 h (Figure 2B). No bacteria were detected at 24 h after inoculation due to a lower detection limit (6 Log_10_ CFU/mL) of the FISH method. In one gut model, 2 to 3 Log_10_ units of *S. aureus* were present after 48 h, in all three vessels (Figure 2B). Such variations in the survival could be influenced by indigenous faecal microflora and dietary habbits of the donors who contributed faecal samples for the study.

*S. aureus* colonies recovered on the selective agar were confirmed by PCR amplification using oligonucleotide primers specific to *S. aureus* specific genes. Interogation of the J. Craig Venter Institute Comprehensive Microbial Resource (http://cmr.jcvi.org) confirmed that *efb* (extracellular fibronogen binding protein) and *spa* (*Staphylococcal* protein A) genes were present only in the sequenced *S. aureus* strains. PCR products *c*. 0.5 and 1.5 kb representing *efb* and *spa* respectively, confirmed that the recovered colonies were indeed *S. aureus* (results not shown).

### Impact of *S. aureus* survival on the colonic microbiota composition

**Figure 3:**
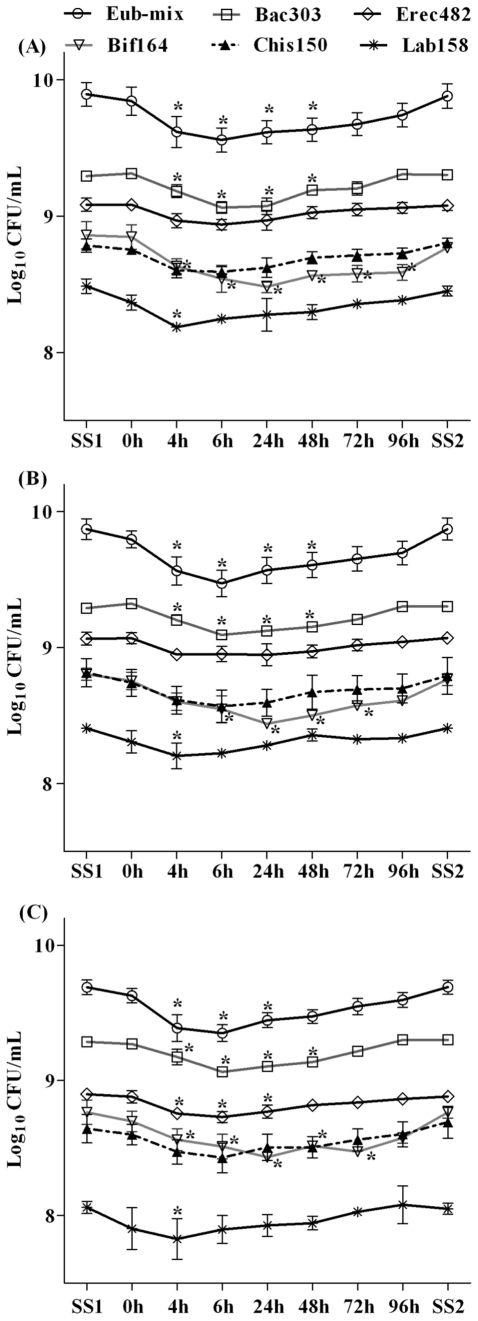
Major colonic bacterial groups detected by FISH in the culture broths recovered from the vessel 1 (A), vessel 2 (B), and vessel 3 (C) of the colonic model before (SS1) and after (0, 4, 6, 24, 48, 72, and 96 h and SS2) inoculation of *S. aureus*. Results are reported as means (Log_10_ CFU/mL) of the data of three colonic models ± standard error of mean. For each colonic model, measurements were performed in triplicate at SS1 (days 11, 12 and 13) and SS2 (days 21, 22 and 23). ^*^ *P*<0.05.

The effect of *S. aureus* survival on the 6 major colonic commensal bacterial groups, were determined for each vessel at SS1 (prior to the inoculation of *S. aureus* into colonic models) and 0, 4, 6, 24, 48, 72, and 96 h post inoculation as well as at SS2 by FISH. In all three model vessels, *Bacteroides* group (Bac303) was the predominant group at SS1, followed by *Eubacterium rectale*/*Clostridium* cluster XIVa (Erec482), *Bifidobacterium* (Bif164), *Clostridium histolyticum* (Chis150), and *Lactobacillus* (Lab158) (Figures 3A, 3B and 3C). A different behaviour was determined for the levels of *Bifidobacterium*, *Bacteroides*, *Lactobacillus* and total bacteria (Eub-mix) in all three vessels and *Eubacterium rectale*/*Clostridium* cluster XIVa for V3 (Figures 3A, 3B and 3C). FISH analysis showed that *Bifidobacterium*, a predominant health-promoting genus of the human gut microbiota [22], [23], decreased significantly from 4 to 96 h post inoculation (P<0.05) in V1, 6 to 72 h post inoculation (P<0.05) in V2, and 4 to 72 h post inoculation (P<0.05) in V3. At SS2, the bifidobacterial counts reverted back to the levels found in SS1.

*Bacteroides*, another common genus of human gut microbiota involved in colonisation resistance to enteric pathogens [24], also decreased significantly from 4 to 48 h post inoculation (P<0.05) in all the three vessels and counts reverted back to the normal levels at SS2 as found in SS1. *Lactobacillus* counts decreased significantly only at 4 h post inoculation (P<0.05) and reverted back to normal levels there onwards in all three vessels. Total bacterial counts decreased significantly from 4 to 48 h post inoculation (P<0.05) in V1 and V2, and 4 to 24 h post inoculation (P<0.05) in V3. Total bacterial counts reverted back to normal levels at SS2 as found in SS1. A significant decrease in the *Eubacterium rectale*/*Clostridium* cluster XIVa counts were determined at 4 to 24 h post inoculation (P<0.05) in V3 only and no significant differences in the counts were found in V1 and V2 throughout the experiments. *Clostridium histolyticum* did not show any significant modification in counts at SS1 and post inoculation periods in all three vessels.

### Impact of *S. aureus* survival on the SCFA production in the colonic models

**Figure 4:**
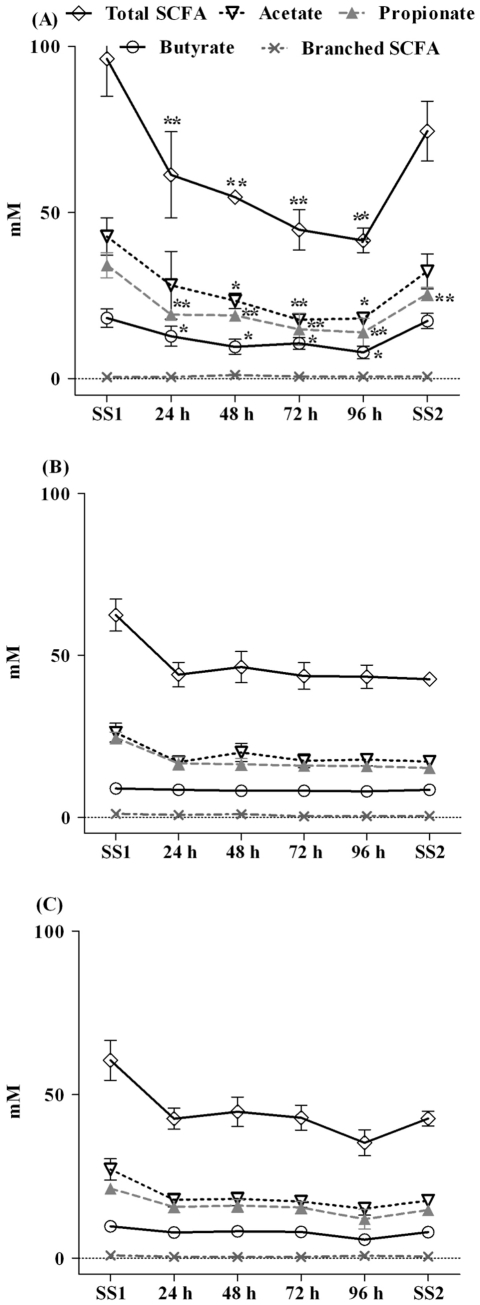
Short-chain fatty acids concentrations in the culture broths recovered from the vessel 1 (A), vessel 2 (B), and vessel 3 (C) of the colonic model before (SS1) and after (24, 48, 72, and 96 h and SS2) inoculation of *S. aureus*. Results are reported as means (mM) of the data of three colonic models ± standard error of mean. For each colonic model, measurements were performed in triplicate at SS1 (days 11, 12 and 13) and SS2 (days 21, 22 and 23). ^*^ *P*<0.05; ^**^ *P*<0.01.

Profiles of SCFAs concentrations prior to the *S. aureus* inoculation (SS1) and during different time periods of post inoculation quantified by gas chromatography are shown in Figure 4. Acetate was the predominant SCFA detected during SS1 and through out different time periods of post inoculation, followed by propionate and butyrate in all three vessels. Significant differences in SCFA profiles were detected only in V1(Figure 4A), whereas changes in the SCFA profiles were insignificant during SS1 and different time periods of post inoculation in V2 and V3 (Figure 4B and 4C). Acetate concentrations decreased significantly from 48 to 96 h post inoculation (P<0.01), whereas decreases in concentrations at 24 h and SS2 were not significant in V1. Propionate levels decreased significantly from 24 to 96 h post inoculation and even at SS2 (P<0.01) in V1. Similarly, butyrate concentrations also decreased signifcantly from 24 to 96 h post inoculation (P<0.05) in V1. Total SCFA levels also decreased significantly from 24 to 96 h post inoculation (P<0.01). The difference between SS1 and SS2 in V1 was not statistically significant (Figure 4A). None of the changes in concentrations of acetate, propionate, butyrate, and total SCFAs in V2 and V3 were significant at any time point, when compared to SS1 (Figure 4B and 4C).

## Discussion

*S. aureus* is able to colonise multiple niches within humans. In doing so, the host is at significantly increased risk of developing disease. Colonisation of the human nares is a well studied risk factor which can predispose an individual to subsequent infection with the same strain [25]. Similarly, *S. aureus* rectal colonisation imparts an increased risk of disease [8]. Interestingly, USA300 *S. aureus* strains, which are responsible for a current outbreak of community acquired disease in the United States, colonise the rectum at rates higher than the nose [26]. A recent study to determine *S. aureus* genetic traits associated with these observed higher rectal carriage rates was inconclusive [27]. Thus it would be very interesting to study the effect that these strains have on gut microbial ecology.

The human intestinal tract is a highly complex ecosystem; whose diverse microbiota profoundly affects health of the host. It provides digestive functions, modulates host metabolism and stimulates angiogenesis, development of lymphatic tissue and the mucosal immune system [28]. Importantly, it can limit infection of the gut by other bacteria. This network of interactions is believed to stabilise the structure of the microbiota population and inhibit colonisation by invading pathogens [29]. Molecular interactions between different members of the microbiota, which prevent establishment of pathogen colonisation, remain to be determined. However, some pathogens are able to disrupt this colonisation resistance and infect the gut [29]. Similarly, the mechanisms that pathogens use to overcome these barriers, formed by the intrinsic microbiota, are poorly understood.

The intestinal microflora is able to ferment plant materials, such as celluloses, starches and sugars, which have been consumed by the host. Mammals lack the necessary enzymes to split such compounds and rely on microbial fermentation to convert indigestible carbohydrates and oligosaccharides into SCFAs, a process which occurs mainly in the proximal colon [30]. In the human colon, acetic, propionic and butyric acids predominate. Of these, butyrate has been particularly well studied because of the effects that it can have on gut health. Metabolism of butyrate represents the preferred energy source for colonocytes and is responsible for synthesis of many key components of the intestinal epithelium [31], [32].

SCFAs such as acetate, propionate and butryate are weak acids with bacteriostatic and bactericidal properties which, in part, depend on the physiological status of the bacteria and the physico-chemical characteristics of the external environment. They can influence the pattern of gene expression in bacteria. Studies on *Salmonella typhimurium* and *Salmonella enteritidis* have shown that SCFAs specifically downregulate genes of the *Salmonella* pathogenicity island (SPI)-1 [33], resulting in supressed invassion of epithelial cells. Conversely, butyrate has been shown to enhance expression of virulence associated genes in enterohaemorrhagic *Escherichia coli* [34].

In addition to direct effects on pathogenic bacteria, SCFAs are able to induce pathogen resistance in host cells [30]. Butyrate treatment of human epithelial cells has been shown to induce resistance to bacterial invasion by *Campylobacter jejuni* or *S. aureus* [35], [36]. Furthermore, butyrate treated epithelial cells produce increased levels of antimicrobial peptides and nitric oxide [36].

Our study demonstrates that *S. arueus* is able to survive the microbial environment of the human gut. Addition of *S. aureus* to the model to mimic infection, resulted in a significant, transient decrease in the numbers of eubacteria and specifically *Bacteroides*, *Bifidobacterium* and *Lactobacillus/Enterococcus* species in vessels 1 and 2, which simulate the proximal and transverse colons, respectively. In vessel 3, which reflects conditions in the distal colon, a broader impact on the microbiota was observed; this involving significant decreases in all of the genera or species measured, except for *Clostridium histolyticum*. The mechanisms by which *S. aureus* is able to colonise the human gut remain poorly understood, however it is now well recognised that a healthy resident microbiota provides good protection against invading pathogens. Given the findings of this study, it can be presumed that *S. aureus* is able to inhibit these bacteria in order to acquire a niche. The eventual loss of detectable levels of *S. aureus* from the model is assumed to be the result of washout from the continuous culture system which is constantly fed with growth medium.

Decreased butyrate in V1 is presumed to result from the altered microbiota, some members of which carry out the conversion of complex molecules to SCFAs. The effect that butyrate has on *S. aureus* remains unknown. While butyrate is bactericidal for some species, it has recently been reported to have no effect on *S. aureus* at neutral pH. However, SCFAs possess increased potency at low pH and any effect of butyrate on *S. aureus* physiology in conditions of the proximal colon (pH 5.5), remains to be determined. Furthermore, decreased butyrate concentrations in the human colon could result in increased localised inflammation, which is believed to facilitate pathogen colonisation [37]–[40].

The complex, beneficial interplay between the host and its microbiota can be interupted by pathogenic bacteria. However any invading pathogen must perturb this delicate association in order to overcome resistance and subsequently colonise and persist within its host. In this study we have demonstrated that *S. aureus* is able to disrupt the normal microbiota of the human colon. The physiological adaptations made by the pathogen in order that it can colonise the gut, remain to be determined.

## Materials and Methods

### Bacteria and Growth conditions

The routinely used laboratory strain *S. aureus* SH1000 [41] was grown on Brain Heart Infusion (BHI) (Oxoid) agar at 37°C.

### Three-stage continuous culture colonic model system (human colonic model)

The three-stage continuous culture model of the human colon comprised of three glass fermenters of increasing working volume, simulating the proximal (V1, 280 mL), transverse (V2, 300 mL) and distal colonic areas (V3, 320 mL). The three fermenters were connected in series, which fed into each other sequentially and finally overflowing into a waste vessel. All vessels were kept at 37°C by means of a circulating water-bath. The pH was controlled and held at 5.5 (V1), 6.2 (V2) and 6.8 (V3) and the system was kept anaerobic by continuously sparging with oxygen free nitrogen gas (15 mL/min). Faecal healthy human samples were collected on site, kept in an anaerobic cabinet (10% H_2_, 10% CO_2_, 80% N_2_) and used within a maximum of 15 min after collection. This experiment was carried out in triplicate using faecal samples from three different volunteers (one faecal donor for each experimental set up). After obtaining verbal informed consent, a standard questionnaire to collect information regarding the health status, drugs use, clinical anamnesis, and lifestyle was administrated before the donor was ask to provide a faecal sample. The University of Reading ethics committee exempted this study from review because no donors were involved in any intervention and waived the need for written consent due to the fact the samples received were not collected by means of intervention. None of the volunteers had received antibiotics or probiotics for at least 3 months before sampling, or steroids or other drugs with a proven impact on gut microbiota composition over the preceding 12 weeks. A 1∶5 (w/w) dilution in anaerobic PBS [0.1 mol/L PBS (pH 7.4)] was prepared and the samples were homogenized in a stomacher (Seward, Worthing, UK) for 2 min. Each vessel was inoculated with 100 mL faecal slurry. Total system transit time was set at 36 h. V1 was fed by means of a peristaltic pump with complex colonic model medium as previously reported [21]. Following inoculation, the colonic model was run as a batch culture for a 24 h period in order to stabilise bacterial populations prior to or the initiation of medium flow. After 24 h (T0) the medium flow was initiated and the system was run for eight full volume turnovers to allow steady state to be achieved (SS1). At SS1, samples were obtained on three consecutive days to confirm steady state status by SCFA and fluorescence *in situ* hybridisation (FISH) based analyses of the microbiota. After the establishment of SS1, V1 of the colonic model was inoculated with *S. aureus* SH1000 (*c*. 2×10^10^ CFU/ml) as a single dose, suspended in colonic model media. Three samples were taken from each vessels of the colonic model at SS1, 0, 4, 6, 24, 48, 72 and 96 h after inoculation with *S. aureus* for viable plate counting and FISH analysis of major colonic bacteria. Samples were also collected on three consecutive days as described for SS1 for a further full eight volume turnovers upon which steady state 2 (SS2) was achieved. Samples for FISH were fixed immediately in 4% paraformaldehyde as previously described [42]. Another set of samples collected at SS1, 24, 48, 72, and 96 h after *S. aureus* inoculation as well as at SS2 were centrifuged at 13000 g and the supernatant stored at −20°C for gas chromatography analysis.

### Enumeration of *S. aureus* on selective agar

Samples (100 µl) of the experimental sets at times SS1, 0, 4, 6, 24, 48, 72, and 96 h after infection as well as at SS2 from all three vessels of the *in vitro* colonic model were plated onto BHI agar containing 0.01% (w/v) potassium tellurite as a selective agent at different dilutions (from 10^2^ to 10^9^ CFU/ml) in triplicate for each time point to measure bacterial growth. Dilutions were prepared using phosphate buffered saline (PBS). The lowest dilution that had bacterial colonies growing on it was used to calculate CFU/mL. As all dilutions were carried out in triplicate, the mean of their log_10_ was calculated (Log_10_ CFU/mL  = n×50× dilution factor; n  =  the number of colonies counted).

### Species- specific PCR analysis

A random selection of single colonies from selective agar plates was inoculated into 10 ml BHI and grown overnight at 37°C. One mL of overnight culture was harvested and DNA samples extracted using the DNeasy Blood and Tissue kit (Qiagen). Extracted bacterial DNA was quantified using a NanoDrop ND-1000 Spectrophotometer (NanoDrop 116 Technologies, Wilmington, DE, USA) and stored at −20°C. The primers (synthesised by Eurofins, Germany) used and the PCR conditions were described in Table 1.

**Table 1:** Primers and PCR conditions for colony screening.

Gene	Oligonucleotide sequences (5′ to 3′)	PCR product size	PCR conditions
*efb*	CGTCAACAGCACATATGAGCGAAGGATACG GCAACGATTGAACTCGAGTTTAACTAATCC	*c*. 0.5 kb	1 min at 95°C; 30 cycles:1 min at 95°C, 1 min at 59°C,1 min at 72°C, and finally 2 min at 72°C
*spa*	CTAGGTGTAGGTATTGCATC CGCTGCACCTAAGGCTAATG	*c*. 1.5 kb	1 min at 95°C; 30 cycles:1 min at 95°C, 1 min at 51°C,1 min at 72°C, and finally 2 min at 72°C

### Enumeration of major colonic bacterial populations by Fluorescence *in Situ* Hybridization (FISH)

FISH experiments were performed as previously described [42]. All probes were Cy3-labelled and synthesized by Sigma Aldrich (Sigma-Aldrich, UK). Table 2 gives the details of the probes used in this study. The composition of hybridization and wash buffers depends on the rRNA-targeted oligonucleotide probe as reported in probeBase (http://www.microbial-ecology.net/probebase) and was used accordingly.

**Table 2:** Oligonucleotide probes used in this study and hybridization conditions for FISH analysis.

Short Name	Target genus	Sequences (5′ to 3′)	Pre-treatment	Temperature (°C)		Reference
				Hybridizing	Washing	
Bac303	Most *Bacteroides* sensu stricto and Prevotella spp.; *all Parabacteroides; Barnesiella viscericola and Odoribacter splanchnicus*	CCAATGTGGGGGACCTT	None	46	48	[44]
Bif164	Most *Bifidobacterium spp*. and *Parascardovia denticolens*	CATCCGGCATTACCACCC	None	50	50	[45]
Erec482	Most members of *Clostridium* cluster XIVa; *Syntrophococcus sucromutans,* *(Bacteroides) galacturonicus* and *(Bacteroides) xylanolyticus, Lachnospira pectinschiza and Clostridium saccharolyticum*	GCTTCTTAGTCARGTACCG†	None	50	50	[46]
Lab158	*Most Lactobacillus, Leuconostoc and Weissella spp.; Lactococcus lactis;* *all Vagococcus, Enterococcus, Melisococcus, Tetragenococcus,* *Catellicoccus, Pediococcus and Paralactobacillus spp.*	GTATTAGCAYCTGTTTCCA‡	Lysozyme	50	50	[47]
Chis150	*Most members of Clostridium cluster I; all members of* *Clostridium cluster II; Clostridium tyrobutyricum; Adhaeribacter* *aquaticus and Flexibacter canadensis (family Flexibacteriaceae);* *(Eubacterium) combesii (family Propionibacteriaceae)*	TTATGCGGTATTAATCTYCCTTT‡	None	50	50	[46]
Sau*	*Staphylococcus aureus*	GAAGCAAGCTTCTCGTCCG	Lysozyme and Lysostaphin	53	53	[48]
EUB338**	Total Bacteria	GCTGCCTCCCGTAGGAGT	None	46	48	[49]
EUB338II**	Total Bacteria	GCAGCCACCCGTAGGTGT	None	46	48	[49]

† R = G/A; ^‡^Y = T/C.

*20% Formamide was incorporated in the hybridization buffer.

**These probes were used together in equimolar concentrations (50 ng/µL) and 35% formamide was incorporated in the hybridization buffer.

### Short chain fatty acids analysis (SCFA) by gas chromatography

Aliquots of 1 mL collected from each vessel in microcentrifuge tubes were centrifuged at 13000 g for 5 min. The supernatants were transferred into fresh microcentrifuge tubes and stored at −20°C until use. Samples were derivatized as previously described [43]. Briefly, the supernatants stored at −20°C were thawed on ice and centrifuged at 13000 g for 10 min. 500 µl of each supernatant was transferred into fresh microcentrifuge tubes and 25 µL of internal standard (2-ethyl butyric acid) followed by 250 µl of concentrated HCl and 1 mL of ether added to each of the tubes. Tubes were vortexed for 1 min and centrifuged at 3000 g for 10 min. The top ether layer was collected and transferred into fresh microcentrifuge tubes. Aliquots (400 µl) of the ether extract were pipetted into a Wheaton vial and then 50 µl of N-tertButyldimethyl silyl N-methyltrifluoroacetamide (MTBSTFA) was added. The vials were sealed tightly by screwing after addition of MTBSTFA and heated at 80°C for 20 min in a water bath. Samples were transferred to Agilent crimp cap vials for gas chromatography analysis. Vials were capped with Crimp top natural rubber/PTFE seal type 7 aluminium silver 11 mm Chromacol caps and sealed using a crimper. The capped vials were left at room temperature for 48 h for derivatization.

Calibration was achieved using standard solutions of derivatized acetic, propionic, *i*-butyric, *n*-butyric, *i*-valeric, *n*-valeric, and *n*-caproic acids as described for test samples. The final concentrations of each standard was 25, 10, 5, 1, and 0.5 mM. The derivatized samples were run through a 5890 series II GC system (HP, Crawley, West Sussex, UK) fitted with SGE-HT5 column (0.32 mm×25 m×0.1 µm; J&W Scientific, Folsom, CA, USA) and flame ionisation detector. Helium was used a carrier gas and delivered at a flow rate of 14 mL/min. The head pressure was set at 10 psi with split ratio 10∶1. Injector, column and detector were set at 275, 250 and 275°C respectively. One micro liter quantity of each sample was injected with a run time of 10 min. Peaks were integrated using the Atlas Lab managing software (Thermo Lab Systems, Mainz, Germany). Fatty acid concentrations were quantified by comparing their peak areas with the standards and expressed in mM.

### Statistical Analysis

All data were analyzed by a One-Way Anova method, using the Tukey post test analysis when overall P of the experiment is below the value of significance (*P<*0.05). Additional paired t-test was applied to understand significance of results of single pairs of data. Analyses were performed using GraphPad Prism 5.0 (GraphPad Software Inc., La Jolla, CA, USA).
